# Multiple Cold Abscesses Without Respiratory Symptoms Revealing Multifocal Tuberculosis: A Case Report

**DOI:** 10.7759/cureus.116475

**Published:** 2026-09-18

**Authors:** Sinthurkka Sathiyanathan, Paransothy Supanchana, Kasun Eranga, Suranga Manilgama, Rakitha Higgoda

**Affiliations:** 1 Department of Internal Medicine, Colombo South Teaching Hospital, Colombo, LKA; 2 Department of Medicine, Faculty of Medical Sciences, University of Sri Jayewardenepura, Colombo, LKA

**Keywords:** cold abscess, extrapulmonary tuberculosis, pleural effusion, soft tissue tuberculosis, xpert mtb/rif

## Abstract

Soft-tissue tuberculosis is an uncommon extrapulmonary manifestation and may be difficult to recognize when respiratory symptoms are absent. We report a 33-year-old, HIV-negative Sri Lankan woman without diabetes or known pharmacological immunosuppression who presented with three months of progressively enlarging, minimally tender superficial collections over the left wrist, right knee, and right ankle, accompanied by anorexia and unquantified weight loss. The swellings had minimal local inflammatory signs. She was anemic and hypoalbuminemic, with elevated inflammatory markers. Separate drainage specimens from all three sites were negative on acid-fast bacillus smear but positive for *Mycobacterium (M.) tuberculosis* complex by Xpert MTB/RIF, without rifampicin resistance; cultures from all three sites subsequently grew *M. tuberculosis* complex. Chest imaging showed bilateral loculated pleural effusions, nodular pleural thickening, and bilateral tree-in-bud nodules. These abnormalities were compatible with probable pleuropulmonary tuberculosis but were not diagnostic. Sputum investigations were negative, and pleural fluid could not be obtained. The case was classified as microbiologically confirmed multifocal soft-tissue tuberculosis with probable pleuropulmonary involvement. All three collections were drained, and first-line antituberculous therapy was initiated. At two months, the lesions remained resolved without recurrence or new lesions; appetite and well-being had improved; weight had increased by 4 kg; erythrocyte sedimentation rate had decreased from 125 to 36 mm/hour; and chest radiography showed near-complete resolution of the pleural effusions. Adherence was satisfactory, and no adverse effects were reported. These findings represented an early clinical and radiological response rather than definitive treatment success.

## Introduction

​​​​​​Tuberculosis (TB) remains an important public-health problem, and extrapulmonary disease may involve almost any anatomical site [1]. A tuberculous cold abscess is a chronic caseous collection characterized by relatively limited acute inflammatory signs and may arise through contiguous or hematogenous spread. Cutaneous and subcutaneous soft-tissue TB is uncommon and may therefore present as a slowly progressive collection with little erythema, warmth, or tenderness [2,3]. Important mimics include pyogenic infection, non-tuberculous mycobacterial infection, melioidosis, nocardiosis, deep fungal infection, and other chronic inflammatory conditions. Bacterial, mycobacterial, and fungal cultures, together with appropriate molecular testing, help distinguish these conditions.

Reports of multiple non-contiguous superficial tuberculous abscesses with concurrent thoracic abnormalities are limited [4]. Multiple lesions may indicate wider disease, while the absence of respiratory symptoms may delay recognition of associated thoracic involvement. When a tuberculous soft-tissue lesion is identified, assessment for pulmonary, pleural, skeletal, and other extrapulmonary involvement is clinically important [5,6]. We describe three microbiologically confirmed, non-contiguous soft-tissue abscesses with radiologically probable pleuropulmonary involvement in a woman who did not report chronic respiratory symptoms.

## Case presentation

A 33-year-old woman resident of Sri Lanka presented with progressively enlarging superficial swellings over the left wrist, right knee, and right ankle for three months. The swellings were not associated with clinically significant pain. She had never lived or travelled outside Sri Lanka. At symptom onset, she experienced an acute febrile illness lasting less than one week that resolved spontaneously. She reported reduced appetite and subjective weight loss. She reported no prolonged evening fever, night sweats, chronic cough, dyspnoea, or known household, occupational, healthcare-associated, or other close contact with a person with active TB. She reported no previously diagnosed comorbidities, previous TB, regular medication use, high-risk sexual exposure, smoking, or illicit drug use.

On examination, she was mildly pale, with a BMI of 20.1 kg/m². She was hemodynamically stable. There was no regional or generalized lymphadenopathy. Cardiovascular, abdominal, and neurological examinations were unremarkable. Respiratory examination showed reduced breath sounds and dullness to percussion over the right lower lung zone, without added sounds.

The swellings measured 2.1 × 1.5 cm over the dorsum of the left wrist, 4 × 4 cm over the dorsum of the right ankle, and 4.5 × 4 cm over the anterior right knee. They were minimally tender, fluctuant, non-transilluminant, and not clinically attached to underlying muscle or tendon. The overlying skin was not warm, red, or erythematous (Figure 1).

**Figure 1:**
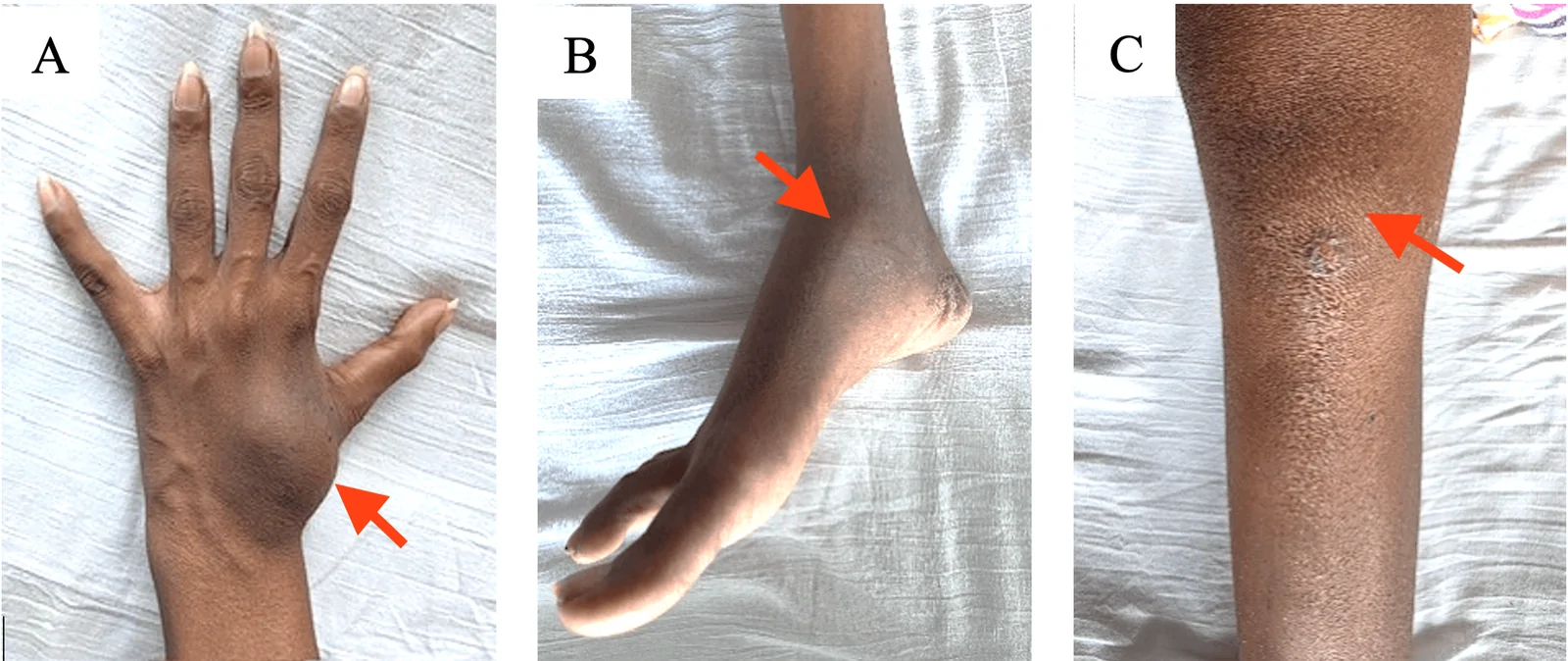
Clinical photographs showing minimally inflamed superficial swellings over the left wrist (A), right ankle (B), and right knee (C).

The principal abnormalities were anemia, elevated inflammatory markers (erythrocyte sedimentation rate (ESR): 125 mm/hour), and hypoalbuminemia. Iron studies were compatible with very mild iron deficiency. The peripheral blood film was predominantly normochromic and normocytic, with a minor microcytic red-cell population, suggesting anemia of chronic disease or inflammation with a possible mild iron-deficiency component. No clinically significant bleeding was identified. Reduced food intake during the period of anorexia supported a possible nutritional contribution. Urinalysis showed no proteinuria. The hypoalbuminemia was therefore considered likely to reflect inflammatory and nutritional factors, although a comprehensive nutritional or gastrointestinal investigation was not performed. A summary of the laboratory results is shown in Table 1.

**Table 1:** Summary of laboratory investigations AFB: acid-fast bacilli; HbA1c: glycated hemoglobin; HPF: high-power field; MTB/RIF: Mycobacterium tuberculosis/rifampicin resistance assay

Investigation	Result	Reference range
Hematology		
White blood cell count	8.63 × 10⁹/L	4–10 × 10⁹/L
Neutrophils	5.94 × 10⁹/L (68.8%)	40%–75%
Lymphocytes	1.66 × 10⁹/L (19.9%)	20%–45%
Eosinophils	0.05 × 10⁹/L (0.6%)	0.5%–5%
Monocytes	0.94 × 10⁹/L (10.9%)	3%–12%
Basophils	0.04 × 10⁹/L (0.5%)	0%–5%
Hemoglobin	9.9 g/dL	12–16 g/dL
Mean corpuscular volume	81.8 fL	83–101 fL
Mean corpuscular hemoglobin	26.1 pg	27–32 pg
Platelet count	241 × 10⁹/L	150–450 × 10⁹/L
Inflammatory, renal, hepatic, and metabolic tests		
C-reactive protein	71 mg/L	<6 mg/L
Erythrocyte sedimentation rate	125 mm/hour	<25 mm/hour
Creatinine	73.1 µmol/L	45–90 µmol/L
Sodium	136 mmol/L	135–145 mmol/L
Potassium	4.9 mmol/L	3.5–5.0 mmol/L
Aspartate aminotransferase	17.2 U/L	10–40 U/L
Alanine aminotransferase	7.4 U/L	7–56 U/L
Albumin	22 g/L	35–50 g/L
Fasting blood glucose	5.6 mmol/L	3.9–5.6 mmol/L
HbA1c	5.5%	<5.7%
Microbiology		
Blood culture	No growth	—
Abscess drainage specimens: Gram stain and routine bacterial culture (all three sites)	No organisms; no growth at all three sites	—
Sputum AFB smear (three specimens)	Negative	—
Sputum Xpert MTB/RIF	Negative	—
Sputum mycobacterial culture	Negative	—
Abscess drainage specimens: AFB smear (all three sites)	Negative at all three sites	—
Abscess drainage specimens: Xpert MTB/RIF (all three sites)	M. tuberculosis complex detected at all three sites; rifampicin resistance not detected	—
Abscess drainage specimens: mycobacterial culture (all three sites)	M. tuberculosis complex isolated from all three sites after five weeks	—
Abscess drainage specimens: fungal culture (all three sites)	No growth at all three sites	—
HIV antibody test	Negative	—
Melioidosis antibody test	Negative	—

Ultrasonography of the right knee showed a subcutaneous collection containing internal echogenic debris, without internal vascularity or a significant joint effusion. Ultrasonography of the left wrist and right ankle also showed hypoechoic lesions in the subcutaneous plane without joint, tendon sheath, or bursal involvement. Plain radiographs of the same anatomical sites showed no bone erosion or periosteal reaction. MRI was not performed because the clinical, ultrasonographic, and radiographic findings did not suggest osteoarticular involvement; limited access to freely available MRI facilities also contributed. Early osteoarticular disease could therefore not be excluded with the same confidence as it could have been with MRI.

Incision and drainage of all three abscesses was performed under local anesthesia. Separate diagnostic specimens were obtained from the left wrist, right knee, and right ankle collections. The purulent material was turbid, whitish-pink, and non-foul-smelling. Results were concordant at all three sites: Gram staining showed no organisms, routine bacterial cultures yielded no growth, and AFB smears were negative. Xpert MTB/RIF detected *M. tuberculosis *complex in all three specimens without rifampicin resistance. After five weeks of incubation on Löwenstein-Jensen medium, mycobacterial cultures from all three sites grew *M. tuberculosis* complex. Fungal cultures showed no growth. No significant tissue was obtained during drainage because the specimens consisted predominantly of fluid and purulent material; histopathological examination was therefore not performed.

Chest radiography showed a homogeneous right lower-zone opacity with blunting of the right costophrenic angle, consistent with a right pleural effusion; there was no obvious cavitation or focal consolidation (Figure 2).

**Figure 2:**
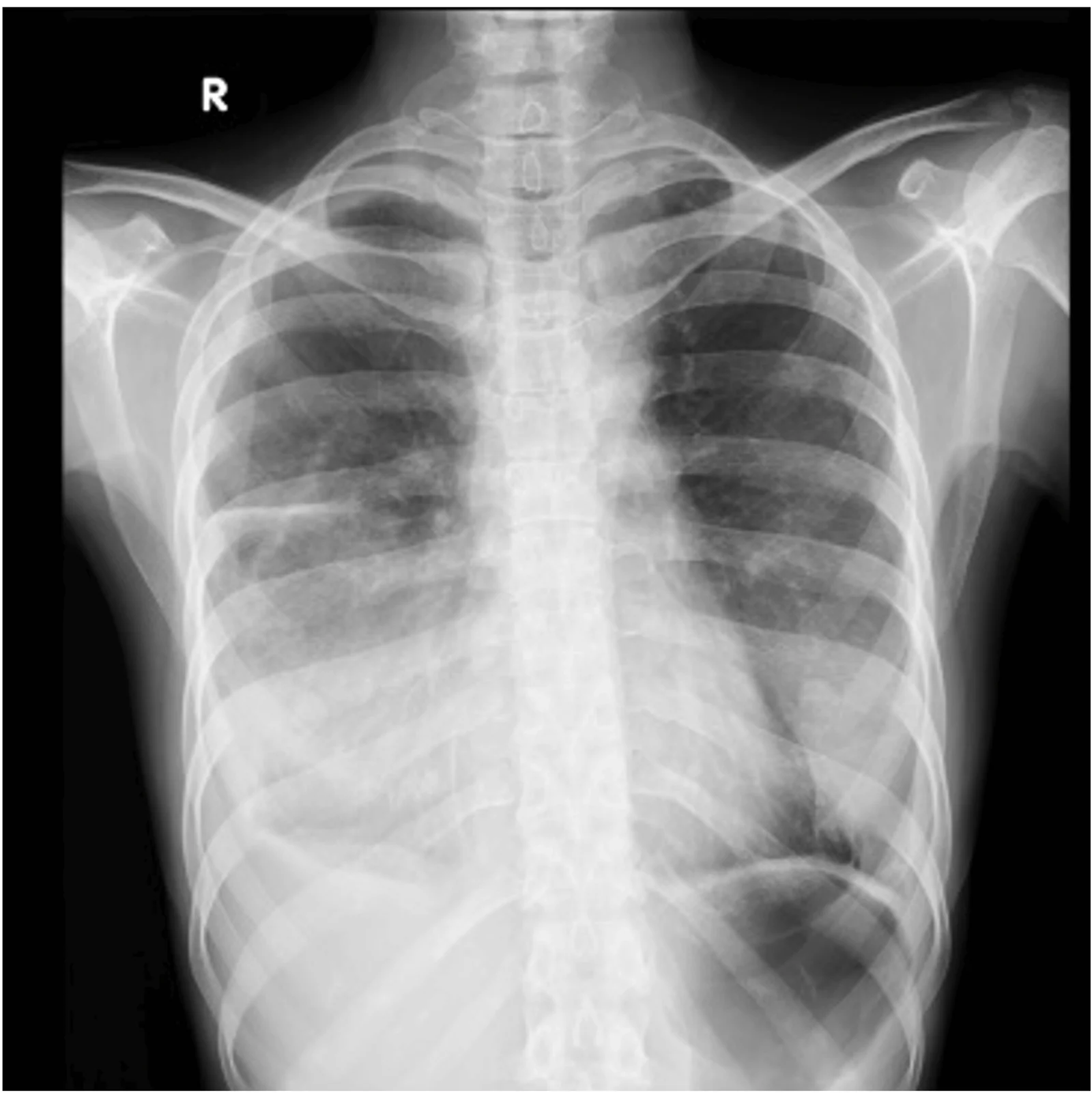
Posteroanterior chest radiograph showing a homogeneous right lower-zone opacity with blunting of the right costophrenic angle, consistent with a right pleural effusion.

Contrast-enhanced computed tomography of the chest, abdomen, and pelvis was formally interpreted by a consultant radiologist. It showed bilateral loculated pleural effusions with nodular pleural thickening and multiple bilateral centrilobular nodules with a tree-in-bud pattern. No cavitation, significant mediastinal lymphadenopathy, or intra-abdominal involvement was reported. In the context of microbiologically confirmed multifocal soft-tissue TB, these abnormalities were compatible with and raised suspicion of pleuropulmonary TB but were not diagnostic. The submitted axial image demonstrates the bilateral loculated pleural collections (Figure 3).

**Figure 3:**
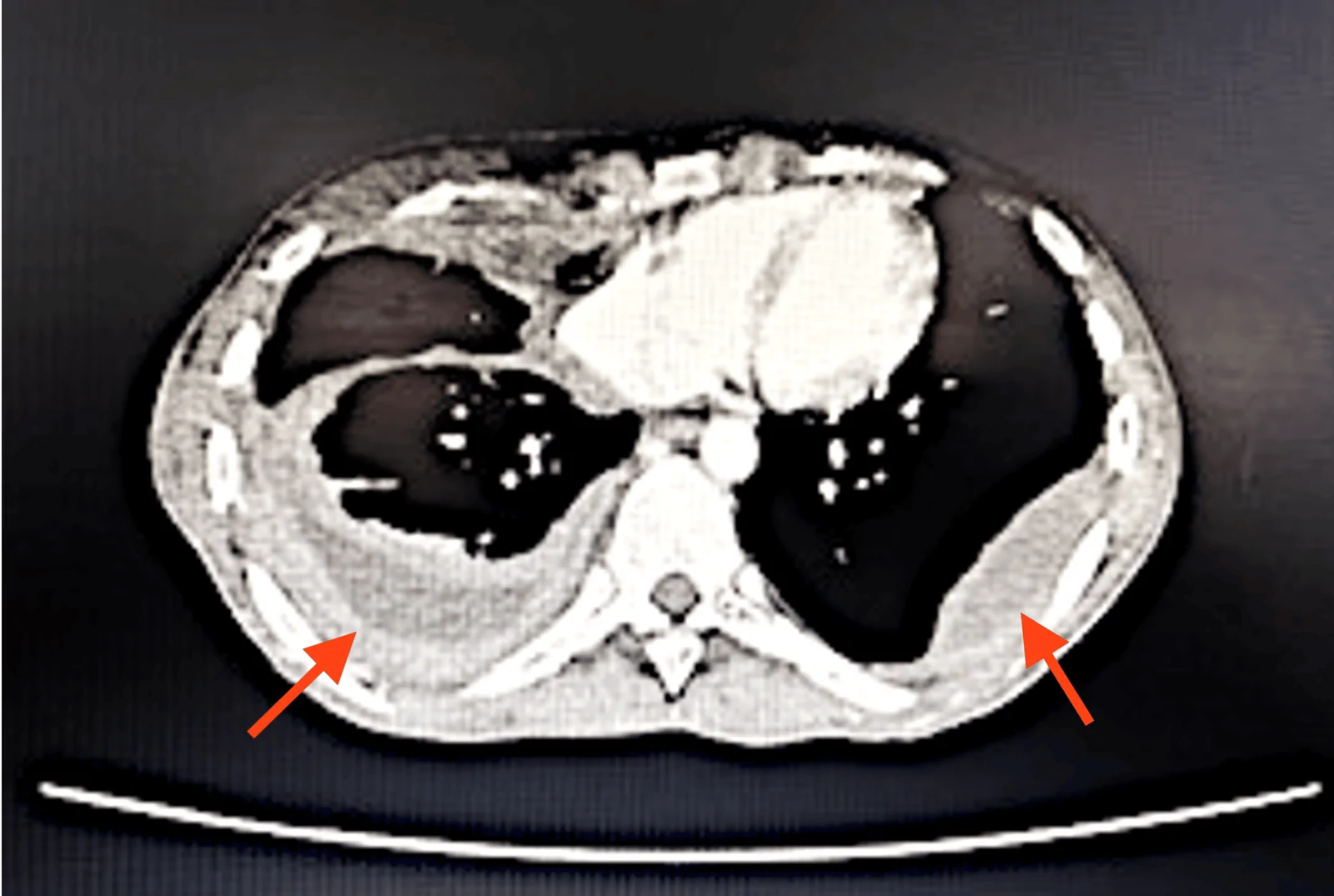
Axial contrast-enhanced CT image at the lower thorax showing bilateral loculated pleural fluid collections, larger on the right

Ultrasound-guided pleural aspiration was attempted but was unsuccessful, and no pleural specimen was obtained. Three induced sputum samples were negative for AFB smear microscopy. Sputum Xpert MTB/RIF and mycobacterial cultures were also negative. Following multidisciplinary review by radiology and respiratory medicine, the working diagnosis was microbiologically confirmed multifocal soft-tissue TB with probable pleuropulmonary involvement. The abnormal respiratory examination, despite the absence of respiratory symptoms, had prompted thoracic imaging.

The patient started first-line therapy with isoniazid, rifampicin, pyrazinamide, and ethambutol as fixed-dose combination tablets, together with pyridoxine. Subsequent culture-based phenotypic drug-susceptibility testing showed susceptibility to all four first-line drugs. A two-month intensive phase followed by a four-month continuation phase with isoniazid and rifampicin was planned. At the most recent documented review, two months after treatment initiation and following completion of the intensive phase, all three abscesses remained resolved, with no recurrent swelling, discharge, sinus formation, or new lesions. Her appetite and general well-being had improved, and a 4 kg weight gain was documented. ESR had decreased from 125 to 36 mm/hour, and repeat chest radiography showed near-complete resolution of the pleural effusions. Adherence was satisfactory, and no adverse effects were reported. These findings were interpreted as an early clinical and radiological response rather than definitive treatment success.

## Discussion

Soft-tissue tuberculosis is an uncommon form of extrapulmonary tuberculosis and may be overlooked because a cold abscess often lacks the erythema, warmth, and marked tenderness expected with an acute pyogenic collection [2,3]. In this patient, three non-contiguous subcutaneous swellings, constitutional symptoms, elevated inflammatory markers, and hypoalbuminemia suggested a chronic systemic process rather than isolated pyogenic abscesses. Ultrasonography localized the lesions to the subcutaneous plane, and radiographs did not show bone erosion or periosteal reaction. These investigations did not demonstrate osteoarticular involvement; however, MRI was not performed, and early osteoarticular disease could not be excluded with equivalent confidence. Previous reports have described isolated and multifocal soft-tissue tuberculosis, but the combination of three microbiologically confirmed superficial lesions and thoracic abnormalities without chronic respiratory symptoms remains unusual [3,4].

The microbiological findings were central to the diagnosis. Abscess drainage specimens from all three sites were negative on AFB smear but positive for *M. tuberculosis* complex by Xpert MTB/RIF, with mycobacterial cultures from all three sites becoming positive on Löwenstein-Jensen medium after five weeks. This pattern is compatible with the paucibacillary nature of many extrapulmonary specimens: a negative smear does not exclude tuberculosis, whereas molecular testing can provide rapid rule-in evidence and simultaneously screen for rifampicin resistance [7,8]. Culture supplied independent confirmation, and subsequent phenotypic testing demonstrated susceptibility to isoniazid, rifampicin, pyrazinamide, and ethambutol.

The extent of disease requires similarly careful interpretation. Bilateral loculated pleural effusions, nodular pleural thickening, and bilateral tree-in-bud nodules were compatible with and raised suspicion for pleuropulmonary tuberculosis in the context of microbiologically confirmed multifocal soft-tissue disease. However, these findings were not diagnostic: tree-in-bud nodules are non-specific, induced sputum smear, Xpert MTB/RIF, and culture were negative, and pleural fluid could not be obtained [6,9]. The most defensible classification was therefore microbiologically confirmed multifocal soft-tissue tuberculosis with probable pleuropulmonary involvement. “Multifocal” describes the three non-contiguous, microbiologically confirmed soft-tissue sites. Hematogenous dissemination was biologically plausible but could not be established from the available evidence. Conversely, the absence of a miliary radiological pattern alone does not exclude disseminated tuberculosis [10]. The route of spread therefore remains uncertain.

No previously diagnosed immunosuppressive disorder was identified: HIV testing was negative, and fasting glucose and glycated hemoglobin were within their stated reference ranges. These findings do not, however, establish normal immune function. Iron studies and the peripheral blood film suggested anemia of inflammation with a possible mild nutritional iron-deficiency component. No clinically significant bleeding was identified. Reduced intake during anorexia, anemia, and marked hypoalbuminemia indicated possible nutritional vulnerability. Urinalysis showed no proteinuria.

The principal alternative diagnoses included pyogenic infection, non-tuberculous mycobacterial infection, melioidosis, nocardiosis, and deep fungal infection. Concordant detection and culture of *M. tuberculosis *complex from all three drainage specimens established the cause of the soft-tissue lesions. Negative Gram staining and routine bacterial and fungal cultures made several competing infections less likely. Melioidosis can also produce multifocal abscesses in tropical settings [11]. The negative melioidosis antibody result and bacterial cultures made melioidosis less likely, although negative serology alone would not exclude it. This case demonstrates the value of submitting chronic, minimally inflamed collections for bacterial, mycobacterial, and fungal studies rather than treating them as routine pyogenic abscesses.

Management combined drainage of all three collections with standard first-line antituberculous therapy and pyridoxine. Drainage provided diagnostic material and treated the symptomatic collections. The extent of surgery should be individualized according to lesion size, persistence, fistulation, underlying structural involvement, and response to antituberculous therapy [12,13]. The planned two-month intensive phase followed by four months of isoniazid and rifampicin is a conventional regimen for drug-susceptible pulmonary and many forms of extrapulmonary tuberculosis when central nervous system and osteoarticular involvement are not demonstrated [8]. In this case, osteoarticular involvement was not evident clinically, ultrasonographically, or radiographically.

This report has several limitations. Histopathological examination was not performed because drainage yielded predominantly fluid and purulent material without significant tissue. MRI was not performed, so early osteoarticular involvement could not be excluded as confidently as it could have been with cross-sectional musculoskeletal imaging. Although the thoracic CT findings were compatible with tuberculosis in the context of microbiologically confirmed multifocal soft-tissue disease, pleuropulmonary involvement was not microbiologically or histologically confirmed because sputum testing was negative and pleural fluid could not be obtained. Follow-up extended only to completion of the two-month intensive phase; definitive six-month treatment outcome and repeat hemoglobin, albumin, and C-reactive protein results were unavailable.

## Conclusions

Chronic, minimally inflamed soft-tissue collections in tuberculosis-endemic settings should prompt consideration of tuberculosis even when respiratory symptoms are absent. When feasible, specimens from each anatomically separate lesion should undergo molecular testing and mycobacterial culture. Multifocal cold abscesses should prompt systematic assessment for pulmonary, pleural, skeletal, and other extrapulmonary disease.
